# Registered nurses’ experiences of communication with patients after the end-of-life breakpoint communication: A pilot interview study

**DOI:** 10.1016/j.ijnsa.2024.100263

**Published:** 2024-11-04

**Authors:** Rebecka Nilsson, Ami Hommel

**Affiliations:** a Malmö University, Department of care science, Jan Waldenströmsg 25, 20506, Malmö, Sweden; b Skåne university hospital, Department of oncology, Tora Kjellgrens gata 27, 214 28 Malmö,Sweden

**Keywords:** Communication, End-of-life breakpoint communication, Fundamentals of care, Nursing, Palliative care

## Abstract

**Purpose:**

Effective nurse-patient communication is demanding and essential when patients’ treatment changes from curative to palliative approach. We aimed to illustrate nurses’ experiences communicating with patients who have undergone end-of-life breakpoint communication.

**Method:**

Six nurses from both haematology and oncology wards at a hospital in southern Sweden were interviewed. The data were analysed using content analysis.

**Result:**

Three themes with a total of eight categories were identified: *Nurses were excluded from the end-of-life breakpoint communication*, which impacted the following communication and the dialogue between nurses and patients afterwards; *the importance of communication,* where the experience of following-up patients, supporting patients and having existential conversations was highlighted; *the nurses’ strategies in the communication*, included experience of using tools, the need for more information and education, and to be human.

**Conclusion:**

Nurses in today's healthcare system need education to provide the care and the communication that patients ask for and are obligated to receive regarding support and information. In the results, nurses suggest and relate their strategies for effective patient communication, which would also aid nurses working in areas other than palliative care. For example, they propose collaborating more with physicians to facilitate better information flow and patient contact. Moreover, reflective tutorials, including spiritual leaders to aid patients’ existential concerns, are beneficial. The need for further research in this area is crucial for the growth and development of nurse-patient communication.

**What is already known about the topic**

• Physicians most often preform the end-of-life breakpoint communication alone with the patient.

• Reactions like fear, anger, and isolation also can arise in patients during the end-of-life breakpoint communication, resulting in their not taking in the information.

• Nurses often feel responsible to have a follow up the end-of-life breakpoint conversation with the patient and describe this as challenging as patients often have many questions, thereby placing much responsibility on the nurses.

Alt-text: Unlabelled box

**What this paper adds**

• A possible theme for future study is whether nurses should participate in the end-of-life breakpoint communication to be able to answer questions from the patients after the physician's end-of-life breakpoint communication.

• Nurses may demand adequate education to manage all kinds of communication

• To be able to provide person-centred care based on holistic care, it might be of value that nurses participate in end-of-life breakpoint communications.

Alt-text: Unlabelled box

## 1 Introduction

The World Health Organization (WHO) defines *palliative care* as an approach that improves the quality of life of patients — adults and children — and their families facing life-threatening illnesses. It prevents and relieves suffering through the early identification, correct assessment, and treatment of pain and other problems, whether physical, psychosocial, or spiritual (WHO, 2020). This definition underscores the hopeful and optimistic role of palliative care in improving the quality of life, even in the face of a life-threatening illness. In the early palliative phase, the disease is incurable, progressive, and deadly, but treatment to prolong life is possible (e.g., antitumoral therapy). The focus of this phase is to prolong life, alleviate symptoms, and give the patient as high a quality of life as possible. In the late palliative phase, treatment is not likely to prolong life, and the goal of care is to provide the best possible quality of life and support to the next of kin (RCC, 2024). The term ‘end-of-life’ is sometimes used to describe the last year, half-year, or last week, and sometimes the point when death will occur within days (Hui et al., 2015). The problem is that the period can be defined only after death. The Swedish National Board of Health and Welfare has defined ‘palliative care in the end-of-life’ as palliative care during the patient's last period of life (NBHW, 2018). The shift to the late palliative phase, or end-of-life care, is commonly referred to as a breakpoint and called end-of-life breakpoint communication not only in Sweden but also in the international literature (Lundquist, 2013; Udo et al., 2018; NBHW, 2018).

When curative treatment is no longer an option, a physician performs a medical assessment to initiate the transition from curative to palliative care. Physicians most often perform the subsequent transition conversation with the patient (Ecarnot et al., 2018; Miller et al., 2022); however, nurses often feel responsible for having a follow-up transition conversation with the patient (Canzona et al., 2018; Warnock et al., 2017). This conversation, while challenging, requires a high level of empathy and understanding. Patients often have many questions, placing much responsibility on the nurses, who often feel it is beyond their competence to answer these questions (Broom et al., 2016; Ecarnot et al., 2018). Previous studies (Miller et al., 2022; Warnock et al., 2017) show that reactions like fear, anger, and isolation can also arise in patients during the end-of-life breakpoint communication, resulting in their not taking in the information. The same can occur if the physician has not provided sufficient information (Ecarnot et al., 2018; Miller et al., 2022). Moreover, after the end-of-life breakpoint communication, nurses sometimes need to assist patients and their next of kin when dealing with denial, anger, and despair resulting from the shock they experience (Canzona et al., 2018). When patients have had difficulties accepting the transition, the conversations are even more difficult for the nurse (Broom et al., 2016).

Nurses are responsible for patient care and assessing, planning, and implementing nursing care (EFN, 2015). Importantly, nurses must be able to communicate with patients in different situations (EFN, 2015); for example, supporting patients experiencing grief and bereavement and paying attention to their last wishes (ICN, 2012). The care should have a holistic approach and focus on physical, psychosocial, spiritual, and cultural aspects (WHO, 2020). To provide person-centred care, which is a care approach that respects and responds to the patient's preferences, needs, and values, the nurse's comprehension of the new focus of treatment is essential. Decisions regarding the direction of care are best achieved through a partnership between patient and nurse (Miller et al., 2022).

The theoretical framework Fundamentals of Care should be seen as a point-of-care nursing theory with the potential to explain and describe the elements of nursing and inform nurses in daily practice (Kitson, 2018).The Fundamentals of Care framework has three core dimensions for the delivery of high-quality fundamental care: a trusting therapeutic relationship between the care recipient and care provider; integrating and meeting a person's physical, psychosocial, and relational needs; and a context of care that is supportive of relationship development and care integration. Trusting relationships are the core of the Fundamentals of Care and are prerequisites for a relationship to be created and thus to meet the patient's needs (Kitson, 2018).

By clarifying to the patient, the transition from curative to palliative care, healthcare professionals and patients create optimal conditions for a patient's remaining time (Miller et al., 2022). Thus, it is essential to ensure that the patient understands the intention of the treatment (Broom et al., 2016). Nurses relate that communication with patients after the transition to end-of-life care conversation is challenging in different ways (Miller et al., 2022; Mishelmovich et al., 2016; Warnock et al., 2017). Despite feeling uncomfortable communicating with the patients after the transition conversation (Broom et al., 2016), nurses deem such conversations important (Broom et al., 2016; Warnock et al., 2017). However, research into nurses' communication experiences with patients after an end-of-life breakpoint communication is limited. In a European setting, some researchers have studied both nurses' and physicians' experiences during and after the end-of-life breakpoint communication (Ecarnot et al., 2018; Miller et al., 2022; Mishelmovich et al., 2016). In a Swedish context, there is also a significant gap in the literature, with only a few studies exploring this phenomenon from the physician's perspective (Lundquist, 2013; Martinsson, 2015; Udo et al., 2018). No studies have yet delved into this from a nursing perspective. Before conducting a more extensive study, this pilot study aimed to illustrate registered nurses' experiences communicating with patients who have undergone breakpoint communication, and highlight identified needs for more research in the Swedish context.

## 2 Methods

### 2.1 Design

The pilot study uses a qualitative method with an inductive approach (Polit and Beck, 2021). Data were collected by individual interviews with nurses at two haematology and one oncology ward at a hospital in southern Sweden. A qualitative content analysis was used to analyse the data (Graneheim and Lundman, 2004; Graneheim et al., 2017). The study was performed following the consolidated criteria for reporting qualitative research (COREQ) checklist to enhance quality and transparency (Tong et al., 2007).

### 2.2 Recruitment

A purposive sampling technique was used (Polit and Beck, 2021). Nurses from haematology and oncology were invited to participate as they could contribute to the aim of the study. The inclusion criteria were nurses with at least one year of working experience and experience with patients who had undergone an end-of-life breakpoint communication. The three wards’ managers selected the nurses according to the inclusion criteria. Nurses who met the inclusion criteria were identified and received an information letter and consent form to sign from their manager. If they were interested in participating, nurses were requested to contact the first author to book an appointment for an interview. In addition, two reminders were sent out to the managers to increase recruitment. In total, seven nurses agreed to participate. All were women who had worked between one and 21 years on either the haematology or the oncology ward.

### 2.3 Data collection

Data were collected through individual face-to-face interviews, except for one telephone interview. A semi-structured interview guide was used. This guide included questions such as 'What is your experience of the communication with patients who have been through the end-of-life breakpoint communication?' and 'what are the difficulties and the opportunities in the communication?' Additional questions about the participants’ wards and work experience were also asked.

A pilot interview was conducted to ensure the understandability of the interview guide and its relevance for the study aim and for practice (Polit and Beck, 2021). The pilot interview led to some changes in the interview guide. Consequently, the pilot interview is not included in the pilot study. The interviews took place at the nurses’ workplace during their normal working hours and were conducted in a private meeting room. The interviews were digitally recorded with the nurses’ consent and transcribed verbatim; no notes were taken during the interviews. The interviews ranged from 20 (the telephone interview) to 55 min (mean 40 min). The nurses did not review the transcribed text before the analysis. The first author conducted all interviews.

### 2.4 Data analysis

The transcribed texts were analysed using inductive content analysis (Graneheim and Lundman, 2004); see Table 1. An inductive approach enables the creation of categories during the analysis and enables the author to see new approaches in content analysis. The analysis was performed at manifest and latent depths (Graneheim and Lundman, 2004; Graneheim et al., 2017). No software was used. The interviews were read several times, and one text was then divided into meaning units according to the purpose. Codes were created based on the meaning units and grouped into subcategories and categories. To find similarities and to gain an overall picture, the subcategories were coloured. The interpretation of categories and subcategories were then divided into themes.

**Table 1:** Example of the data analysis process.

Meaning units	Condensed text	Subcategory	Main category	Theme
As a nurse, you try to follow up and support the patients who are feeling bad mentally after the end-of-life breakpoint communication	Patients who are feeling badly need to be supported.	Support patients	To provide support to the patient	The importance of communication
If you had talked to the physician before the end-of-life breakpoint communication or even better participated at the end-of-life breakpoint communication, then it was easier to answer the patients’ questions.	Communication with the physician or participating at the end-of-life breakpoint communication, gives advantage to answer patients’ questions.	Advantages of participating at the end-of-life breakpoint communication	Impact of not participating at the end-of-life breakpoint communication	The RNs were excluded from the end-of-life breakpoint communication

### 2.5 Ethical considerations

In accordance with the Declaration of Helsinki (World Medical Association, 2022), before interviews began all participants were informed both orally and in writing that participation was voluntary and could be withdrawn at any time without any negative consequences. Before the interviews, all participants signed an informed consent. Further, the interviews were scheduled at the participants ‘convenience, and the data was treated with confidentiality. All material handled confidentially and stored in a locked room and on digital media with password protection.

## 3 Results

**Fig. 1:**
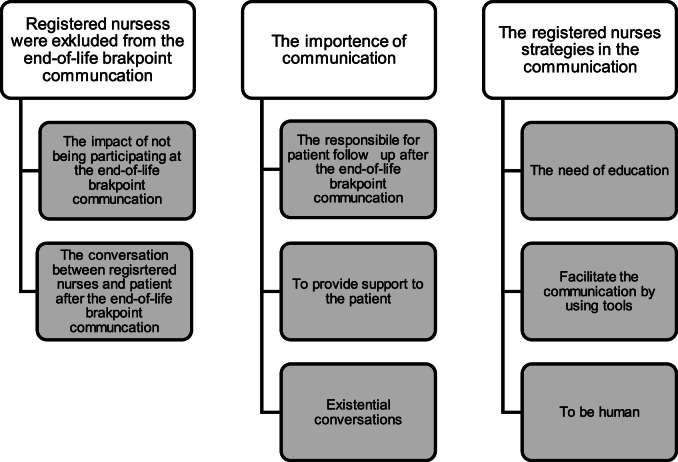
Three themes and eight categories were identified.

Three themes and eight categories were identified and considered to reflect the nurses’ experiences (see Fig. 1). Overall, the themes throughout were “communication” and “conversation.” The communication theme constituted one-way communication, including informative calls from the nurses to the patient. The conversation theme included exchanging communication, thoughts, and feelings between the nurses and the patients. Insert Fig. 1 about here

### 3.1 Nurses were excluded from the end-of-life breakpoint communication

#### 3.1.1 Impact of not participating at the end-of-life breakpoint communication

The nurses reported being rarely involved in the end-of-life breakpoint communication despite wanting to. This was due to various factors, such as no invitation to the transition conversation or lack of time due to responsibility for other patients. Nurses related the importance of participating in the end-of-life breakpoint communication to bring the nursing perspective into the conversation, hear information from the physician, and witness the patients’ reactions:

*I wish that I had participated in the end-of-life breakpoint communication to see the patients’ reactions with my own eyes, to see how their relatives reacted, and to verbatim hear the information the physician provides to be able to add to or explain the concept to the patient. (Nurse 5)*

Nurses and physicians are integral to the patient care process. Nurses have highlighted the consequences of not participating in the end-of-life breakpoint communication, such as not reaching the patient at a deeper level of communication and having the patients retell them the information, which sometimes led to misunderstandings. They have also pointed out that the physicians’ record keeping after the end-of-life breakpoint communication was often too clinical, necessitating the nurses to approach them for more information to clarify and answer patients’ questions. In this collaborative process, nurses consult the physician before talking to the patient to increase their comfort level in the conversation.

#### 3.1.2 The conversation between the nurses and the patient after the end-of-life breakpoint communication

After the end-of life breakpoint communication, nurses said patients often asked them many questions, perhaps due to their accessibility. The nurses found communicating with patients after the transition conversation challenging because they feared giving incorrect or different information from the physician. Nurses stated that although it was acceptable to not always have answers to the patients’ questions, it was essential to give patients the time to communicate after the end-of-life breakpoint communication:

*I don´t need to talk that much. That is something important that nurses should learn. I don´t need to have answers. I just need to be there for the patient. (Nurse 6)*

The nurses were most comfortable answering questions concerning the palliative care unit and advanced care at home. The nurses felt that their working experience enhanced the patients' care, including answering their questions. However, in some situations, they still felt it was difficult. The nurses explained that their communication was adapted to the individual. Sometimes, the patients were aware of their illness and comfortable with the decision, resulting in fewer questions. On the other hand, meeting patients after the transition conversation when the information was neither accepted nor acknowledged was arduous. In such situations, the nurses returned when the patients were more ready to communicate.

Some nurses considered it an advantage that patients brought their relatives to the end-of-life breakpoint communication because they could help the nurses in the communication; that is, to understand what the patients wanted and what they were thinking. Another aspect that affected the conversation was whether the patient and the nurses already had a relationship. The nurses regarded this in two ways. Firstly, it meant that patients sought their consultation rather than the physicians', thus leading to more openness and security. Secondly, it placed added pressure on the nurse's professionalism:

*More difficult emotionally but also easier to have a relationship to the patient before because you have talked to each other previously and have maybe already talked about some of the things that might come up after the transition conversation. Maybe you have talked about what the patient likes to do or what brings the patient happiness, so you as a nurse can use those things in the communication later. (Nurse 6)*

According to the nurses, the patient's reactions and communication output depended on the stages of the transition conversation: from curative to palliative or early palliative to late palliative. For the curative to the palliative conversation, the nurses experienced heavier reactions from patients, often related to the shock reaction during or after the end-of-life breakpoint communication. Consequently, information intake was low, and the transition was dismissed, all of which the nurses found most challenging. Another challenge was when patients needed to talk but could not due to being engulfed by emotions, which the nurses considered problematic as it was beyond the scope of their professional responsibility.

### 3.2 The importance of communication

#### 3.2.1 *The responsible follow up after the* e*nd-of-life breakpoint communication*

All the nurses felt a strong commitment to conducting patient follow-up after an end-of-life breakpoint communication While this was not a professional requirement, the nurses did so, as they considered this part of nursing. However, the nurses experienced lack of education and adequate practical information to follow up with patients after the end-of-life breakpoint communication. The nurses conducted patient follow-ups after transition conversations if requested by the care receivers. The nurses also disclosed that they found it more challenging to conduct follow-ups with younger patients and patients they could relate to personally.

While it was often the nurses who initiated communication after the end-of-life breakpoint communication, they were also open to patients taking the first step. The nurses' follow-up methods were described in two ways. Some aimed to provide familiarity and competence regarding the new focus and availability if patients had any questions, while others immediately asked questions about the patients’ feelings related to the new focus and how they could help them. This flexibility in approach demonstrated the nurses' commitment to patient-centred care:

*After the end-of-life breakpoint communication, I used to go to the patient and check the situation if there were any questions. I wanted to show them that I am familiar with the new focus and that I can support them if there is something they are wondering about or need. (Nurse 4)*

All the nurses explained that their follow-up communication was based on how the physician provided the patient with information during the end-of-life breakpoint communication. If the information had been presented clearly and straightforwardly, it would have been easier for the nurses to communicate with the patients afterwards, thereby reducing the number of questions from patients.

The same occurred when the end-of-life breakpoint communication was conducted too quickly, and the physician needed to give the patients time to ask questions; these questions were then referred to the nurses instead. In such cases, the nurses felt that they could refer the questions to the physician if they were unable to answer them. The nurses explained this situation because they did not have adequate information about earlier conversations with the physicians. Consequently, they were aware they needed to be flexible to conduct patient follow-ups.

The nurses described that the patients accept the information about the transition only after some time, and they often need multiple follow-up conversations. Some patients even needed repeated medical conversations with a physician if they had not accepted or received enough information during the end-of-life breakpoint communication. Regardless, the nurses performed follow-up conversations with the patients, as it was considered a part of nursing and an opportunity to ask the patients for their future wishes.

#### 3.2.2 To provide support to the patient

The nurses experienced that they supported patients differently depending on the patient reaction and need for support. The nurses could adapt the support to the individual situation, and it felt easier to help the patients if the nurses had participated in the end-of-life breakpoint communication:

*As a nurse, you need to be flexible with many roles. Sometimes you are a psychologist, and sometimes you are a hairdresser, adapting to what the patient needs in the moment. (Nurse 5)*

Some patients had a significant need to talk afterwards due to feelings of sadness and hopelessness, while other patients did not because they were already familiar with the new focus of the care. The nurses, in their proactive approach, explained that they supported them in three ways: informing patients about their accessibility, their availability to answer questions anytime, and supporting patients by checking in on them. Some patients needed to have the information from the end-of-life breakpoint communication retold, especially when the transition conversation was experienced as a shock. Other patients were satisfied by the support of holding a hand.

Nurses, with their patience and empathy, sometimes gently prodded patients with the question, “How do you feel?” They saw this as a means of encouraging patients to be open. Other ways of supporting patients were inquiring about their needs, accommodating them, and assuring them that their remaining time would be as comfortable as possible. The nurses also had to support the patients in conversations about the future with their families, which were conversations patients feared. Nurses described meeting the patients with their fears and feelings about the future to process it together.

*It is important not to forget that the patients need support from us nurses. It is not only their relatives that may also be sad and shocked. I think patients need support from others as well as their relatives, and that will be us. (Nurse 2)*

The nurses reported they needed to confirm the reality of the situation to the patients, who wanted validation of their emotions. Although the situation was already emotionally charged for the nurses, it was even more challenging to support patients with whom they already had a relationship. However, it was also seen as an opportunity to better support the patient due to previous knowledge. Nevertheless, the previous relationship made it more difficult for the nurses to distance themselves from the situation. Another difficulty was supporting patients in denial of the gravity of the situation; in such cases, it was deemed best to wait until the patient was ready to communicate. Most nurses reported that it was difficult to support the patients in their situation, despite one nurse who described that it was easier to support the patient when the focus of treatment now was clear.

#### 3.2.3 Existential conversations

Patients frequently had existential (human existence) thoughts and questions after the end-of-life breakpoint communication, which the nurses considered a significant challenge:

*They have many questions about life and death and about what is happening after you die, and how long I they will live. They are really difficult questions because we do not have an answer to that. (Nurse 2)*

Sometimes, the existential questions arose after a while. However, such existential thoughts could disappear following the trauma of the end-of-life breakpoint communication as patients tended to focus on practical matters as a defence mechanism. The nurses conveyed that they felt emotionally affected by the existential questions and thoughts, especially when the patient had been making plans that would now be cancelled due to the transition. In such instances, the nurses explained that maintaining their professional rather than personal roles made it easier for the nurses to manage the situation. They reported their fear of existential conversations due to their lack of education and experience.

Consequently, nurses referred such conversations to the physician instead. However, the nurses highlighted the importance of having such existential conversations, which they viewed as part of nursing. The nurses also described possibilities in the existential conversations to illustrate palliative care earlier to the patients and to create individual conditions based on the patient's wishes. However, it also clarifies where the patient wants to die and their wishes on how end-of-life care should be conducted. Another possibility in existential conversations is to get the patient's mind on something else for a while to reduce anxiety, such as encouraging them to live life as long as possible.

### 3.3 The nurses’ strategies in the communication

#### 3.3.1 The need for information and education

The nurses explained that relevant information and education had made a difference when caring for the patients. However, they felt that they needed more education regarding complicated conversations. They also described how education would not help complicated conversations because these would be difficult regardless. The nurses reported that nursing education did not include how one should have these conversations.

The nurses disclosed that it was problematic that only some of their peers were familiar with what an end-of-life breakpoint communication constituted, resulting in no patient follow-ups later. The nurses requested education not only about dealing with existential conversations but also education on what a transition conversation is and how it should be performed:

*And to talk about death, that is not a subject that people are comfortable to talk about, so it can be difficult and uncomfortable. So, we need both education and practical experience in existential conversations. (Nurse 4)*

One nurse suggested that a spiritual leader could come and lecture on how existential questions could be answered and how one could more easily talk about death.

#### 3.3.2 Facilitate communication by using tools

The nurses used tools to handle communication more easily; consequently, they requested more tools to facilitate improved communication. Moreover, they pointed out that internal discussions about professional difficulties were an effective tool, as was using resources such as a counsellor or a spiritual leader if patients needed them. Other communication tools were more practical, such as sitting on a chair or squatting during a conversation, which could benefit communication by giving the patient experience that the caregiver had more time. Participants also suggested creating guidelines to facilitate better communication with patients, although this was seen as problematic in catering for individual patient needs. Another suggestion was to offer patients a follow-up dialogue with a physician and a nurse to answer questions and for information to be repeated. It was expressed that patients feel comfortable knowing such a follow-up will occur within a few days. Furthermore, more tools were requested for inexperienced colleagues to prepare them more for holding complicated conversations.

The nurses' professional experience is a powerful tool, and by participating in the end-of-life breakpoint communication, they not only share their knowledge but also gain new insights. The nurses highlighted that experiencing the transition with patients multiple times served to increase their communication competency and foster continuous learning. At one hospital ward, a monthly tutorial was used to reflect on patient cases that had impacted the ward or to discuss care-related challenges. Additionally, bi-weekly ethical sessions on death or patient loss were found to be beneficial, providing a platform for intellectual stimulation and engagement.

*It's not just a person who is dying, but someone's mother or wife. I think that we keep too much to ourselves; we usually do that. But you need to reflect. I think that you must talk about it in a group to make everyone feel all right. (Nurse 3)*

#### 3.3.3 To be human

During the conversation with patients after the end-of-life breakpoint communication, the nurses experienced a sense of duality in being professional and personal when supporting patients and answering their questions. One nurse described the situation as professionally tricky, which they sought to navigate as effectively as possible. Another nurse claimed that patients were more trusting if the nurse showed compassion and emotion, highlighting the powerful impact of their actions on patient trust:

*You will get more trust from patients when they can feel that the person beside their bed is not a robot. There is a human too. (Nurse 1)*

The nurses described that they allowed themselves to cry and show hopelessness in front of the patient to show humanity:

*Generally, I think that it should be okay to be personal. I think that we gain something from it. It will give the patients the little extra instead of just walking in and out. (Nurse 2)*

## 4 Discussion

We found that the participating nurses’ experience of communicating with patients after the end-of-life breakpoint communication could be understood by considering three predominant themes: the nurse's exclusion from the transition conversation, the importance of communication, and the nurses’ communication strategies. This aligns with other researchers (Broom et al., 2016; Ecarnot et al., 2018) describing challenges, as patients often have many questions after the transition conversation, placing much responsibility on the nurses, who often feel it is beyond their competence to answer them. Furthermore, we showed that these nurse participants missed important information about the new treatment focus, a crucial aspect that determines the patient's need for support, by not participating in the end-of-life breakpoint communication due to a lack of invitations. For nurses to adapt care from a holistic perspective, they need to be well-informed about the nursing measures (Kitson et al., 2018) and the new treatment focus in these cases.

According to psychosocial and relational dimensions, the nurse should establish communication by listening to the patient and showing empathy (Kitson et al., 2018). Several nurses in the current study highlighted one reason to participate in the end-of-life breakpoint communication – to bring the nursing perspective into the conversation, which was confirmed by researchers in several other studies (Canzona et al., 2018; Ecarnot et al., 2018). Often, nurses can flag a patient's poor condition even before a physician has considered having a transition conversation (Canzona et al., 2018; Ecarnot et al., 2018). The nurses requested an opportunity to plan the transition conversation collectively with a physician. Commonly, end-of-life breakpoint communication are inadequately planned, as not all the relevant professions are invited to attend, which is not optimal. (Warnock et al., 2017).

All nurses conducted patient follow-ups after the end-of-life breakpoint communication through a sense of responsibility and as an essential part of nursing. They described challenges regarding physicians providing patients information and subsequent patient reactions, which aligns with the findings of other studies (Canzona et al., 2018; Miller et al., 2022; Broom et al., 2016). If the nurses had participated in the end-of-life breakpoint communication, negative consequences could have been reduced. In another study (Warnock et al., 2017), nurses’ experiences of not having a patient follow-up conversation were due to time restraints or patients being placed in a multi-bedded bedroom. Nurses in the current study highlighted having timely patient follow-up conversations jointly with physicians to support the patient. According to Kitson (2018), nurses should create a safe environment for the patient, which could be promoted through such follow-up meetings.

The nurses highlighted the need for more education about complicated conversations, including existential conversations, which was corroborated by findings from (Canzona et al., 2018; Broom et al., 2016; Mishelmovich et al., 2016). Patients’ reactions can make the conversations challenging, and nurses need to be able to deal with such situations. In the current study, this lack of knowledge did not discontinue the conversation, which was found in Abbaszadeh et al. (2014), especially conversations about death. However, nurses found it problematic to answer questions about death and existence. Groebe et al. (2019) claims such topics have long been taboo amongst health professionals, offering a potential explanation. The nurse's reference to the importance of daring to stay in the conversation and meet the patients’ fears, a crucial step in effective patient care, aligns with findings from Miller et al. (2022). The nurses were emotionally affected by existential talk, resulting in their responding professionally and personally. We, as well as other researchers (Mishelmovich et al., 2016), indicated that showing a more human side rather than a professional side could help the nurse facilitate existential conversations. An existential conversation should not be forced (Groebe et al., 2019). Through a good relationship, a nurse can more easily assess the patient's need for care, meet the patient in complicated conversations, and dare to ask the patient questions (Miller et al., 2022; Mishelmovich et al., 2016; Werkander Harstade et al., 2018).

We found challenges in the communication end-of-life breakpoint communication Patients often asked the nurses questions that the latter sometimes felt difficult to answer. According to Muntlin Athlin et al. (2018), nurses should dare to stay with the patients to ascertain their needs and show them dignity. Nurses should be able to provide knowledge and inform patients about their care. When planning care, they can learn about the integration between patient and nurse (Muntlin Athlin et al., 2018). Nurses work closely with patients, and the Fundamentals of Care highlight the meeting with the patient and the integration between the patient and the nurse to provide holistic care (Kitson, 2018). To provide person-centred care based on holistic care, nurses must participate in the transition conversation and receive adequate information and education to manage communication, emphasizing the need for continuous learning in the healthcare profession.

### 4.2 Strengths and limitations

The pilot study was based on a small sample (*N* = 6), which could have implications regarding its trustworthiness and could be seen as a limitation regarding the possibility of a conclusion since we did not reach data saturation. However, the material from the interviews was rich and nuanced. Nevertheless, a study that includes more hospitals and participants is needed to confirm the results.

One of the six was a telephone interview, which was the shortest of all the interviews, but it still gave more transcribed material than one more extended face-to-face interview. Thus, the telephone interview is not considered to have affected the result. Regarding recruitment, it was considered a strength that the first author was present at unit meetings and sent out multiple reminders to augment the recruitment. The first author works as an oncology nurse and has a certain preunderstanding of the aim. However, she did not work at the wards where the informants were included. Using gatekeepers distanced the first author from the recruitment.

Moreover, using a semi-structured interview guide and doing the content analysis in collaboration distanced the first author's pre-understanding. As there are always different ways to interpret a text, there is a specific risk of subjectivity (Graneheim and Lundman, 2004). The authors did not have a relationship with the included participants in the study.

## 5 Conclusion

Nurses may need education and support to provide the care patients request. In addition, it may be essential for nurses to feel comfortable in their caregiver role so that communication with patients does not stop. Further, they may need to engage with patients about their existential thoughts. Nurses in the current study suggested numerous solutions to the challenges they faced with communication. For example, they proposed collaborating more with physicians to facilitate better information flow and patient contact.

Moreover, reflective tutorials, including spiritual leaders to aid patients’ existential concerns, may be beneficial. This would not only help them better conduct such conversations but also increase the quality of the communication. Such suggestions can be integrated into staff training in all the different wards of somatic care to increase the nurse's confidence.

## CRediT authorship contribution statement

**Rebecka Nilsson:** Writing – original draft, Methodology, Formal analysis, Conceptualization. **Ami Hommel:** Writing – review & editing, Validation, Supervision, Methodology, Conceptualization.

## Declaration of competing interest

The authors have no conflicts of interest to declare.
